# Piezoelectric MEMS Mirror with Lissajous Scanning for Automobile Adaptive Laser Headlights

**DOI:** 10.3390/mi13070996

**Published:** 2022-06-25

**Authors:** Bin Xu, Yao Ji, Kai Liu, Jinhua Li

**Affiliations:** 1 Department of Mechanical Engineering, Sichuan University, Chengdu 610065, China; bin_xu@outlook.com (B.X.); jiyao@stu.scu.edu.cn (Y.J.); 2 Department of Electrical Engineering, Sichuan University, Chengdu 610065, China; kailiu@scu.edu.cn

**Keywords:** piezoelectric, MEMS mirror, laser scanning, automotive, laser headlight

## Abstract

The emergence of smart headlights with reconfigurable light distributions that provide optimal illumination, highlight road objects, and project symbols to communicate with traffic participants further enhances road safety. Integrating all these functions in a single headlight usually suffers from issues of bulky multi-functional add-on modules with high cost or the use of conventional spatial light modulators with low optical efficiency and complex thermal design requirements. This paper presents a novel laser headlight prototype based on biaxially resonant microelectromechanical systems (MEMS) mirror light modulator for mapping blue laser patterns on phosphor plate to create structured white illumination and tunable road projection. The proposed headlight prototype system enables reconfigurable light distribution by leveraging laser beam scanning with fewer back-end lens and simple thermal design requirements. Built with thin-film lead zirconate titanate oxide (PbZrTiO_3_) actuators, the MEMS mirror achieved high-frequency biaxial resonance of 17.328 kHz, 4.81 kHz, and optical scan angle of 12.9°. The large mirror design of 2.0 mm facilitates more refined resolvable projection pixels, delivers more optical power, and provides moderate optical aperture to possibly serve as the common spatial light modulator of headlight and the light detection and ranging (LiDAR) towards all-in-one integration. The carefully designed bi-axial resonant frequency improves the device’s robustness by offsetting the lowest eigenmode away from the vehicle vibration. By establishing the laser headlight prototype systems of both 1D and 2D scanning modes, a mathematical model of laser modulation and MEMS electrical control principles of Lissajous scanning are proposed to tune the projection pattern density and shapes. It laid the foundation for developing a laser scanning control system with more complex project functions and prompting the application of MEMS for compact headlight system that addresses night driving visibility, eliminates glare effect, and renders interactive projection capabilities.

## 1. Introduction

Driving safety and vehicle intelligence enhancement are the main technological evolution trend of automotive industry. Since the accident rate for driving at night is two times higher than that for driving at daytime [1], night driving safety ranked as the top safety topic with great concern and improving driving visibility at night has been considered a key solution for this issue. The controversial demands of drivers and pedestrians for maximum and refined spatial road illumination but minimal glare have motivated engineers to develop advanced automotive lighting systems. Adaptive driving beam (ADB) is a sophisticated driver assistance technology for vehicle headlights to improve road and traffic safety at night. It provides drivers with high contrast and brightness visibility at night while eliminating glare that causes temporary blindness for other drivers [2,3,4]. Unlike traditional headlights that manually switch between low and high beam modules [5], the ADB system is built on an automotive computer vision system that regulates the spatial illumination based on the cruising speed and the traffic situation (e.g., the presence of oncoming traffic, pedestrians, traffic marks, or vehicles ahead traveling in the same direction) [6]. With recent rapid advances in computer vision recognition and solid-state light source of headlight modules, it has been technically feasible for processor-control ADB systems to serve as a crucial component of automotive electronics [7]. In parallel, recent booming progress in artificial intelligence and sensor fusion have evolved ADB systems towards highlighting road objects and projecting symbols to communicate with other traffic participants. The driver is allowed to obtain information about the vehicle’s surroundings by illumination marks, further enhancing road safety [6,7,8,9]. As the complexity of the external environment and road conditions increases, vehicle smart headlights are constantly developing. Implementing all functions in a single headlight typically requires multiple light modules with high and low resolution, which are unfriendly to the installation space, appearance design, and cost of headlights.

Some commercial ADB optics employ light-emitting diode arrays to digitally regulate the projected light patterns. It is also possible to use a sliding shutter in front of the lamp to partially block the light path with mechanical movement [5]. However, due to the limited tunable degrees of freedom in such systems, the projected light is restricted by the shape without fine area adjustment capability, which even leads to over-blocking of road signs or nearby pedestrians. In other cases, arrays of liquid crystal on silicon (LCoS) [10] pixels or digital micromirror devices (DMD) [11] have recently been utilized as the spatial light modulators (SLMs) [12] for ADB systems with a high degree of freedom of programming. Since the SLMs share similar optics with picture projectors, they can create more complex illumination patterns for additional functional features such as projecting navigation or warning mark on the road. However, these SLM-based optics suffer problems of costliness. Moreover, due to the modulate mechanism of global lighting of pixels followed by masking, the optical power of the SLMs that has been shadowed will rapidly heat up the headlight module [13]. As a result, the optical efficiency decreases as the pixel shadow area increases, leading to a necessity for complex thermal design to prevent the ADB system from overheating damage [14,15].

As an alternative candidate for the SLMs of ADB, MEMS 2D laser beam scanners (LBSs) show superior optical efficiency and system simplicity by directly reflecting the modulated blue laser and generating configurable illumination patterns through phosphorous materials and single lens [16,17,18,19]. Compared with the LCoS and DMD, MEMS LBSs operate with a pixel-level light-on-demand mechanism, which greatly reduces waste heat generation [20]. The much simpler module thermal design and fewer back-end lens array requirements greatly reduce the costs. Asari et al. [5] proposed a headlight system based on a piezoelectric MEMS scanner, but the relatively small mirror aperture design restricted the achievable resolution limit and the maximum delivered optical power of the system. Moreover, their low-stiffness meandering suspensions design of mirror support structure lower the 1st eigenmode frequency to below 1 kHz, which is critical for MEMS to easily react with external random vibrations of the vehicle [21]. Danov et al. [22] presented a large aperture MEMS mirror with high-frequency Lissajous scan and headlight module. However, the necessity of vacuum package increases the device cost and introduces a side effect of reflection at the cover glass interface. The mathematics for laser modulation and electrical control principle of Lissajous MEMS scan has yet to be clarified. In addition, since MEMS LBSs have been mostly adapted as the emission modulator for LiDAR [21,23], they also enable a promising technical possibility to embed smart headlights and LiDAR within a single module by sharing a common MEMS mirror as a spatial light modulator for all-in-one integration of a compact and cheaper module. However, to the best of our knowledge, the reported studies are still far from optimal for this purpose.

**Figure 1:**
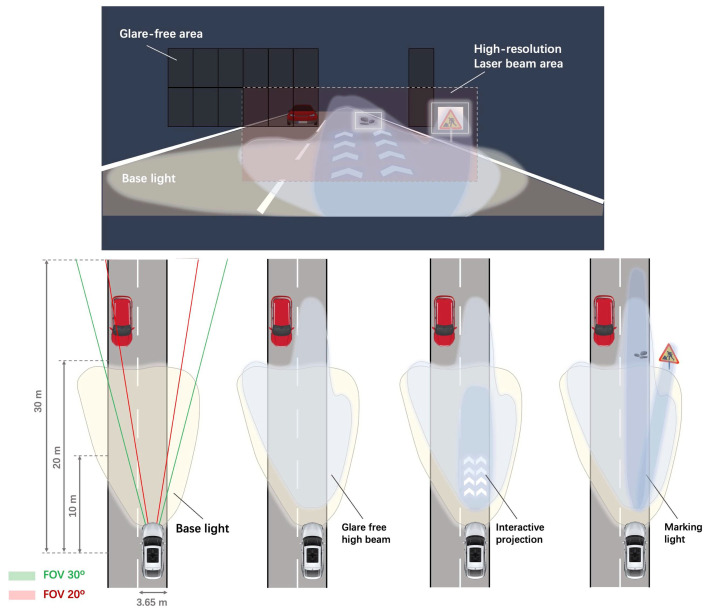
The schematic of the MEMS scanning adaptive laser headlights with different road illumination functions.

To address the issues, this paper proposed a novel proof-of-principle headlight prototype based on a MEMS LBS with both large mirror aperture, high-frequency bi-axial resonance design and simple piezoelectric actuation at ambient condition. A 2D MEMS mirror with lead zirconate titanate (PZT) thin-film actuation operates by synchronizing with a blue laser diode (LD) to draw a Lissajous pattern and projects programmable patterns onto a phosphor plate for color conversion. The large mirror aperture of 2.0 mm not only facilitates more refined resolvable dots and delivers more optical power, but also provides an optimal mirror size to integrate LiDAR and headlights in a compact system by sharing a common MEMS as a spatial light modulator. The carefully designed bi-axial frequency of Lissajous scanning improves the device reliability by offsetting the lowest eigenmode away from the vehicle random vibration (<2 kHz) [5]. Particularly, a mathematical model of the laser modulation and electrical control principles of MEMS are well established for the Lissajous scan. It paves the way to develop a MEMS laser scan optical system of more complex functions and prompt the application of MEMS SLMs for a compact ADB system that addresses nighttime driving visibility, eliminates glare effects, and renders interactive projection capabilities, as shown in Figure 1.

## 2. Piezoelectric MEMS Laser Beam Scanner

**Figure 2:**
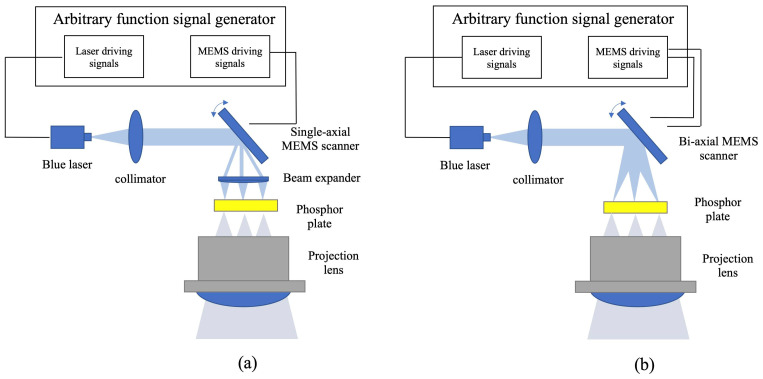
Flowchart of the laser headlight prototype based on (**a**) 1D MEMS laser beam scanning and (**b**) 2D Lissajous MEMS laser beam scanning mode.

Figure 2 shows the flowchart of the laser headlight prototype system based on 1D and 2D Lissajous MEMS laser beam scanning that has been developed in this work. Based on a MEMS scanner, the laser headlamp prototype utilizes simple projection optics to achieve reconfigurable light distribution, simple module thermal design, and fewer back-end lens requirements that significantly reduce costs. As illustrated in Figure 2a, a blue laser is collimated by a collimator and emits a collimated beam, which is spatially modulated by a single-axial MEMS scanner. The reflected beam is then linearly expanded by a beam expander to generate a 2D pattern projected forward onto a phosphor plate that converts the blue light into a high brightness white pattern. Finally, the white pattern is projected forward through a projection lens. Unlike the laser headlight prototype based on a single-axial MEMS scanner, in the laser headlight prototype based on a bi-axial MEMS scanner, the collimated laser beam is spatially modulated by the bi-axial MEMS scanner to generate a programmable 2D pattern directly as shown in Figure 2b. Both the laser driving signals and the MEMS mirror driving signals are generated by an arbitrary function signal generator. The intensity of the laser is modulated in synchronization with the MEMS scanner to create dynamic patterns. Therefore, in the subsequent discussion, we will detail the design requirements of the MEMS scanner, the fabrication process, the experimental characterization process, and discuss the mathematical model of the MEMS mirror scan trajectory.

### 2.1. MEMS Scanner Design

The main actuation principles of the MEMS laser scanner include electrostatic actuation [24], electromagnetic actuation [25], electrothermal actuation [26], and piezoelectric actuation [27]. The use of piezoelectric actuation in this MEMS mirror design is mainly based on the relatively large actuation forces of low driving voltage and simple device architectures [28,29]. In contrast to conventional actuators, the piezoelectric actuator operates on a simple stacking thin-film PZT structure without using complex deep trench etching for non-planar comb-drive electrode or wet process of metallic coil electroplating. It also facilitates angular amplification mechanics to drive larger mirror size at low power consumption but large scanning angle and high resonant frequency [29].

The illumination and projection of laser headlight systems are achieved by tuning the resolution and shape of the scanning Lissajous patterns, which are strictly controlled by the fast-axis and slow-axis frequency of the MEMS mirror [30]. To meet the resolution requirements of projection pattern in the headlight system (VGA or above), the MEMS mirror needs to be oscillated at least 16 kHz [31]. Meanwhile, due to the mathematical essence of the Lissajous trajectory and the design boundary conditions of vehicle random vibration, it is also necessary to match the bi-axial scanning frequency to realize arbitrary control of Lissajous patterns, which requires a slow-axis frequency over 4 kHz.

**Figure 3:**
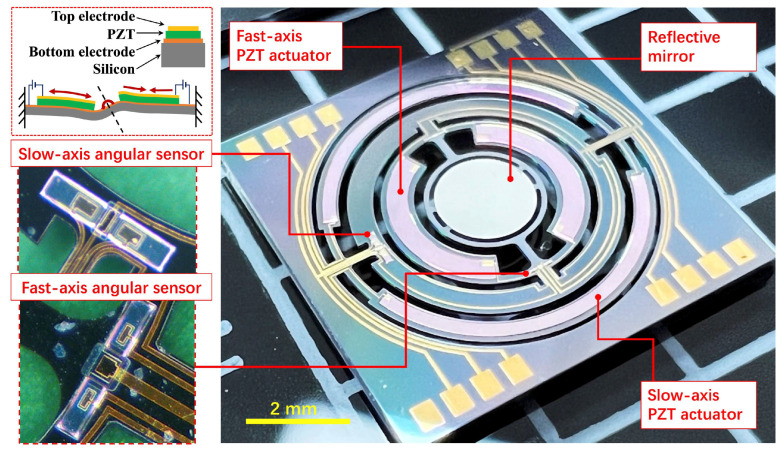
The structure of piezoelectric MEMS mirror with built-in PZT actuator and angular feed-back sensor for bi-axial Lissajous scanning.

A photograph of the as-fabricated MEMS mirror is shown in Figure 3. The entire chip is built on a silicon-on-insulator (SOI) wafer of a 100-μm-thick device layer with a 2-μm-thick PZT thin film formed on it. The central mirror plate of Φ = 2 mm is supported by a pair of torsion beams (width of 160 μm, length of 680 μm). The pink areas in the image correspond to the PZT parts with top-electrode metallization and angular feedback sensors. In detail, the actuator structures of semilunaris shape contain a couple of PZT unimorph actuators to excite the resonance of the mirror plate in a torsional mode and tilt around the axis of torsion beams. The intermediate circular gimbal frame that contains the mirror plate and the actuators is supported by a pair of straight beam suspensions of 160 μm in width and 550 μm in length. The combination of the out-of-plane resonant rotation in the mirror plate and gimble frame enables 2D laser beam scanning. As shown in the zoom-in graph, the angular feedback sensors with a pair of typical piezoelectric transducers are positioned near the end of torsion beams to maximize the displacement-to-electrical signal transfer.

As the actuation mechanism shows in the close-up image of Figure 3, the top electrodes of actuator are loaded with differential AC voltages with frequency modulation, while the bottom electrodes are grounded. In this case, one of the PZT actuators generates compressive stress in the silicon frame surface while the opposite one produces tensile stress, thus bending the beam to twist the torsional bar. The torque of torsional bar transfers to the mirror plate and finally excites mechanical resonance of fast-axis scan in a horizontal direction. The slow-axis motion in the orthogonal scanning direction is driven by the PZT actuators of outer frame in the same manner as the fast-axis counterpart. The gimbal frame of the scanner is actuated by the same twist mechanism around the slow-axis orthogonal to the fast horizontal direction.

### 2.2. Fabrication Process

**Figure 4:**
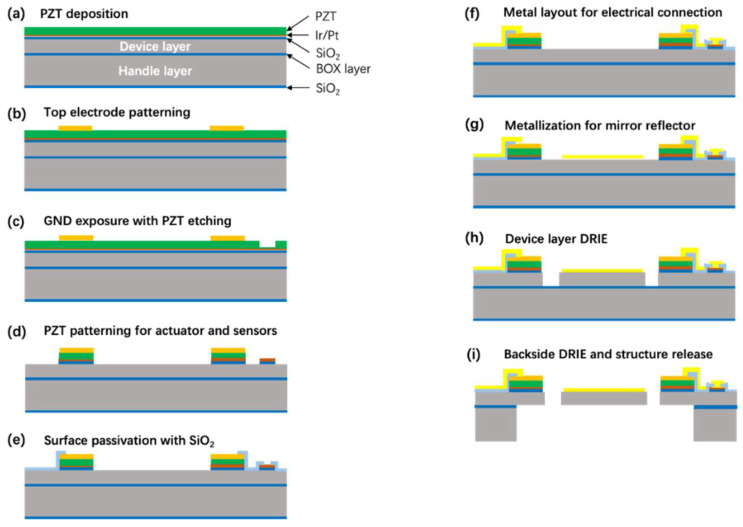
Microfabrication process flow of the piezoelectric 2D MEMS mirror. (**a**) PZT deposition. (**b**) Top electrode patterning. (**c**) GND exposure with PZT etching. (**d**) PZT patterning for actuator and sensors. (**e**) Surface passivation with SiO_2_. (f) Metal layout for electrical connection. (**g**) Metallization for mirror reflector. (**h**) Device layer DRIE. (**i**) Backside DRIE and structure release.

The device fabrication started from silicon-on-insulator (SOI) wafers of 100-μm-thick device layer, 2-μm-thick buried oxides (BOX), and 450-μm-thick handle layer with both sides of 500 nm-thick surface oxides, as illustrated in Figure 4. (a) Bi-layer of Ti/Ir (30 nm/100 nm) was deposited on the wafer surface as an adhesion promotor and bottom electrode for piezoelectric actuation. Subsequently, a 2-μm-thick PZT thin-film was formed through a sputtering process. (b) Then, Ti/Au layer (30 nm/150 nm) was lithography patterned by a lift-off process to form the top-electrode of PZT and electric connection pad for wire bonding. (c) The bottom electrode was partially exposed to GND connection via a PZT dry etching with reactive gas of SF_6_. (d) The piezoelectric actuators and sensors area were further patterned by etching through PZT/bottom electrode/surface oxide layers and extending the top-side dry etching into the silicon device layer. (e) An additional 300-nm-thick SiO_2_ layer was then sputtered and partially removed to work as a surface insulation with exposed geometric area for subsequent electric connection and further silicon device layer etching. (f) Gold wire connection was laid out to form a conductive connection between the PZT top-/bottom-electrodes and the electric pads. (g) Another mask was utilized to define geometric structure area to extend topside etching of the device layer by the deep reactive ion etching (DRIE) until the BOX stop layer. By this step, the basic device structures of the mirror plate, torsion bars, and piezoelectric actuators were formed. (h) With photoresist passivation and protection of the as-fabricated fine structures, the handle layer of SOI substrate was patterned to release the device movable structures via a back-side DRIE process. Finally, the BOX layer was selectively removed to release the structures.

### 2.3. Device Characterization

**Figure 5:**
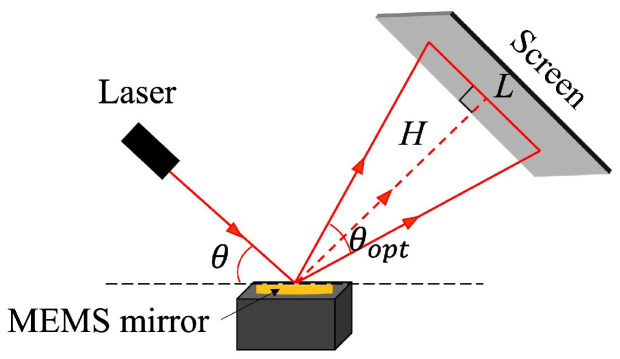
The schematic drawing of the device characterization experimental setup.

Figure 5 shows a schematic drawing of the experimental setup. The experimental setup consists of a MEMS mirror, a laser source (λ = 650 nm), and a receiving screen with an adjustable angle. In addition, it also includes power supply, arbitrary function signal generator, and oscilloscope. The MEMS mirror was mounted on the optical workbench, the modulated laser was incident on the reflective mirror plate at an angle of *θ* = 45 degrees, and the angle between the screen and the horizontal plane was manipulated at 45 degrees to ensure that the reflected laser spot was perpendicular to the screen. The relationship between *H*, $\theta_{opt}$, and *L* is expressed by the following equations:

(1) $$\theta_{opt}=2\tan^{-1}(L/2H)$$

where *H* = 480 mm, $\theta_{opt}$ and *L* denote the vertical distance between the screen and the mirror, the optical scanning angle of the MEMS mirror and the length of projected laser lines, respectively.

**Figure 6:**
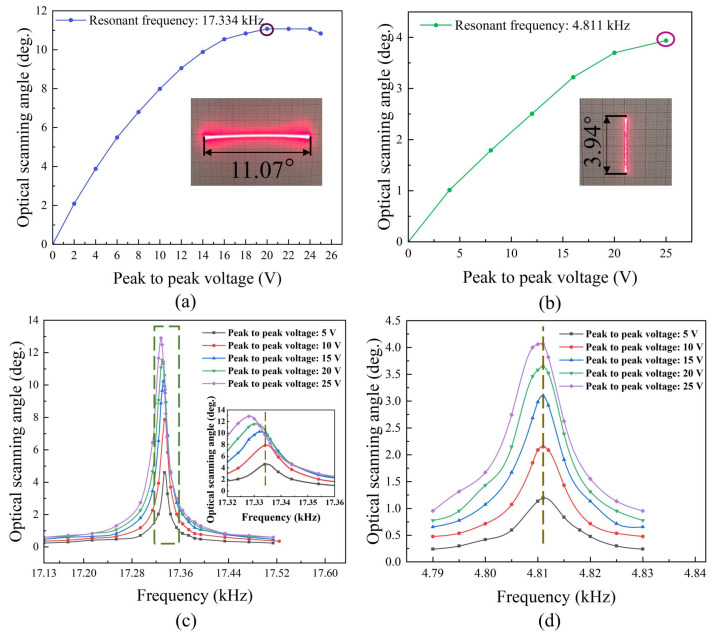
The schematic of the resonant frequency of MEMS mirror. (**a**,**b**) The relationship between the driving voltage of the fast and slow axis versus $\theta_{opt}$ at a constant resonant frequency, respectively. (**c**,**d**) The relationship between the central resonant frequency of the fast and slow axis versus $\theta_{opt}$ under various driving voltages.

In order to evaluate the $\theta_{opt}$ of the fast-axis and slow-axis scanning of MEMS mirror, the piezoelectric film was driven at the resonant frequency of 17.334 kHz and 4.811 kHz, respectively, by sweeping the actuation voltage from 5 Vpp to 25 Vpp, as shown in Figure 6a,b. The fast-axis showed a maximum $\theta_{opt}$ = 11.07° at a driving voltage of 20 Vpp, while the slow-axis scanning showed a maximum $\theta_{opt}$ = 3.94° at a driving voltage of 25 Vpp. Both fast-axis and slow-axis scanning performed non-linear behavior over 20 Vpp actuation. As depicted in Figure 6c,d, further study of the non-linearity was performed by analyzing the relationship between bi-axial resonant frequency and the $\theta_{opt}$ at the peak-to-peak voltage ranging from 5 V to 25 V. At each actuation voltage, the scanning angle was maintained at maximum by manually adjusting the actuation signal frequency. The fast-axis displayed more obvious central resonant frequency shift than that of the slow-axis. Therefore, the fast-axis and slow-axis scanning achieved $\theta_{opt}$ of 12.9° and 4.06° with resonant frequency of 17.328 kHz and 4.810 kHz, respectively, at driving voltage of 25 Vpp.

**Figure 7:**
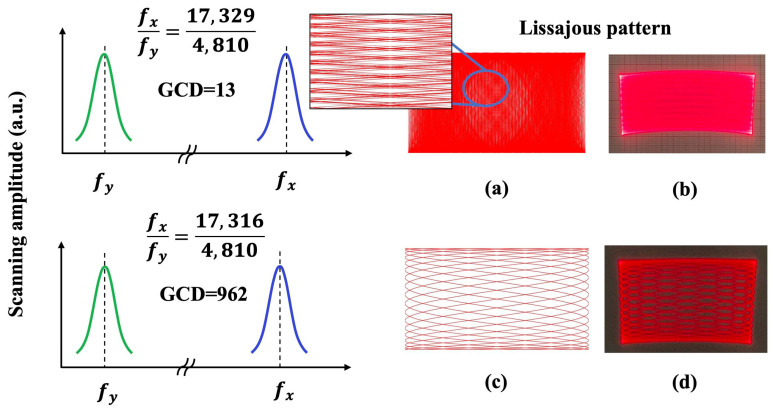
The relationship between the scanning frequency of the MEMS mirror and the projected Lissajous pattern. Note that $f_{x}$ and $f_{y}$ infer the resonant frequency of the fast-axis and the slow-axis of MEMS mirror, respectively. The density of the Lissajous pattern decreases as the great common divisor (GCD) of bi-axial scanning frequencies ($f_{x}$ and $f_{y}$) increases. (**a**,**b**) $f_{x}$ = 17,329 Hz, $f_{y}$ = 4810 Hz, GCD = 13. (**c**,**d**) $f_{x}$=17,316 Hz, $f_{y}$ = 4810 Hz, GCD = 962. Where (**a**,**c**) were generated by algorithm simulations, (**b**,**d**) were generated by actual laser patterns projection.

A Lissajous scanning trajectory can be obtained by simultaneously operating the fast and slow axes of the mirror based on sine signal waveforms at different frequencies as follows [18]:

(2) $$\{\begin{array}{c} X=A_{x}\sin(2\pi f_{x}t+\varphi_{x})\\ Y=A_{y}\sin(2\pi f_{y}t+\varphi_{y}) \end{array}$$

where X and Y indicate the position of a laser scanning beam over a scanning time. *A* is the scanning amplitude corresponding to the peak-to-peak voltage, $f_{x}$ and $f_{y}$ are the scanning frequency of the fast and slow axis, respectively, and t is the scanning time. φ corresponds to the phase for each axis, in this work, $\varphi_{x}$ and $\varphi_{y}$ were both set at 0 degrees. The fast-axis and the slow-axis of the MEMS mirror were actuated simultaneously to achieve Lissajous scanning and guide the laser beam to draw Lissajous patterns. Figure 7 indicates a schematic illustration of the relationship between the scanning frequency of the MEMS mirror and the Lissajous trajectory. In this experiment, two different sets of scanning frequency combinations were tested, and the corresponding Lissajous patterns were obtained by algorithm simulations and actual MEMS laser patterns projection. Since the incidence of laser beam was not perpendicular to the MEMS mirror and the incident stereo angle changes during the biaxial mirror rotation, the projected Lissajous trajectory shows a downward bending trend and thus a sector-shaped distortion, as shown in Figure 7b,d. The Lissajous patterns are built on a strict mathematical relationship between the scanning frequency of MEMS mirror. The greatest common divisor (GCD) of scanning frequencies ($f_{x}$ and $f_{y}$) of the MEMS mirror directly determines the density of the Lissajous pattern, whose pattern density is increased by decreasing the GCD of the scanning frequencies. As a result, when the GCD is very small, the scanning trajectory of the Lissajous patterns could not be clearly observed and presented as highly dense 2D patterns are shown in Figure 7a,b. The tunability of the scanning line density and shape size of the Lissajous pattern laid the foundation for the headlight systems with dynamic spatial light modulation capability, as will be discussed later.

**Figure 8:**
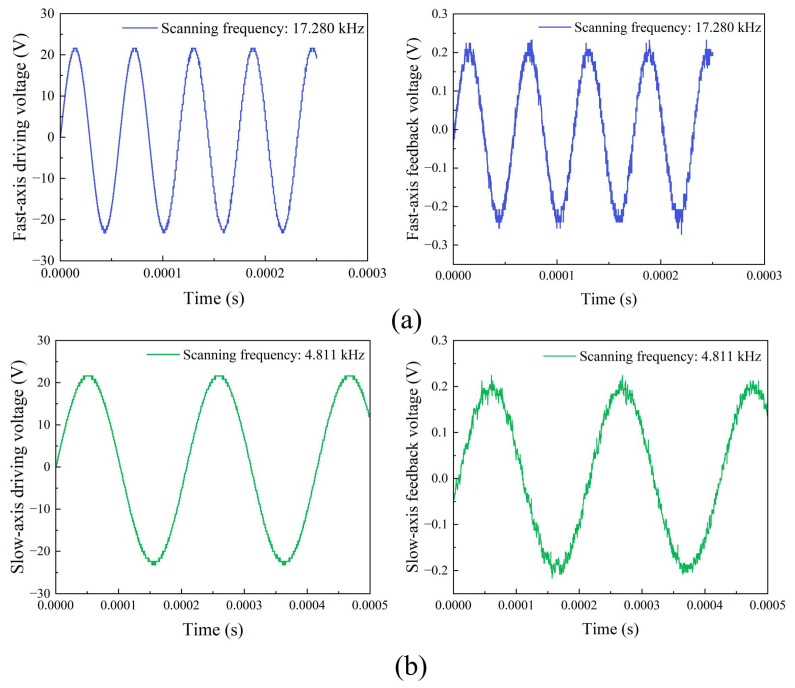
Driving voltage signal (Left) and feedback voltage signal from the built-in angular sensor (Right) for (**a**) fast-axis scanning (**b**) slow-axis scanning.

As stable image display of Lissajous laser scanning system heavily relies on the synchronization between laser modulation and mirror scanning behavior, a pair of thin-film piezoelectric transducers were designed near the end of each torsion bar to monitor the mirror motion by picking the maximum deflection and obtaining largest gain of voltage signal generation. As illustrated in Figure 8, the couple of angular sensors were designed to detect the mirror resonant frequency, scanning amplitude, and phase delay to enable feedback control of laser modulation for automated calibration of image generation error with a clear pattern projection.

## 3. Evaluation of Prototype of Laser Headlight Optics

**Figure 9:**
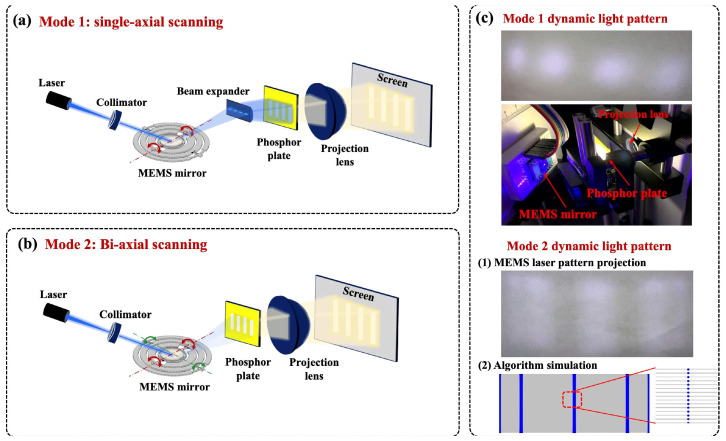
The schematic drawing of the laser headlight prototype optical system architecture with (**a**) 1D scanning mode and (**b**) 2D Lissajous mode. (**c**) The light patterns generated from (**a**,**b**) with experimental and simulation parameters, mode 1: $f_{M}$ = 17,328 Hz, $N_{1}$ = 4, $L_{M1}$ = 69,312 Hz, $N_{f1}$ = 4, mode 2: $f_{x}$ = 17,328 Hz, $f_{y}$ = 4810 Hz, $N_{2}$ = 8, $L_{M2}$ = 69,312 Hz, $N_{f2}$ = 5.

Figure 9 shows a schematic drawing of laser headlight prototype optical system based on the as-designed MEMS mirror SLMs with both 1D and 2D Lissajous scanning, and their dynamic illumination patterns projection. As illustrated in Figure 9a collimated blue laser beam (wavelength of 450 nm, power of 70 mW, beam diameter of 1 mm) was spatially modulated by the MEMS mirror with only fast-axis actuation. Then, the reflected beam passes through a beam expander (plano-convex cylindrical lens with focal length of 6 mm) generating a 2D pattern forward and projecting onto the phosphor plate for white color conversion. Finally, the white patterns were projected onto the screen receiver through a focusing lens (plano-convex lens with focal length of 25 mm). The laser frequency modulation rule of the single-axis scanning optical system is as follows:

(3) $$L_{M1}=N_{1}f_{M}$$

where $N_{1}$ is a positive integer, $L_{M1}$ and $f_{M}$ denote the laser modulation frequency and the scanning frequency of the MEMS mirror, respectively. The scanning trajectory completely repeats every period of $1/f_{M}$.

To generate fringe patterns with different densities, the laser modulation frequency follows the rule in Equation (3). Further, the number of fringes ($N_{f1}$) can be calculated by Equation (4):

(4) $$N_{f1}=\{\begin{array}{ll} N_{1}, & N_{1}=2n+1\\ N_{1}/2+1, & N_{1}=4n\\ N_{1}/2, & N_{1}=others \end{array}$$

where *n* is a natural number. Figure 9c mode 1 shows the dynamic light pattern which is generated by the laser headlight prototype system based on a MEMS mirror with 1D scanning when $N_{1}$ = 4. It is noted that the phase delay of the MEMS mirror causes the captured pattern showing $N_{f1}$ = 4 instead of the theoretically calculated $N_{f1}$ = 3 in Equation (4).

As illustrated in Figure 9b, the laser spots were directly projected forward on the phosphor plate by spatially modulating ethe bi-axial MEMS scanning mode. Variable Lissajous patterns can be generated according to the strict mathematical relationship between the fast and slow axis frequency of the MEMS mirror and the laser modulation frequency. Refined illumination for local area highlights can even be achieved by matching the laser switch-on duration with bi-axial scanning mirror trace. As depicted in Equation (2), if $f_{x}$ and $f_{y}$ are both integers with the greatest common divisor $f_{0}$, then the following relationships can be obtained:

(5) $$\begin{array}{c} \frac{f_{x}}{f_{y}}=\frac{n_{x}f_{0}}{n_{y}f_{0}}=\frac{n_{x}}{n_{y}}\\ \frac{1}{n_{x}}=\frac{1}{f_{x}}f_{0} \end{array}$$

where $1/f_{x}$ is scanning period of the MEMS mirror and $n_{x}/n_{y}$ corresponds to the frequency ratio while both $n_{x}$ and $n_{y}$ are dimensionless constants. When the fast- and the slow-axis of the MEMS mirror are actuated to generate the Lissajous patterns, the scanning trajectory is repeated periodically with $1/f_{0}$, which is the frames per second (FPS) and linearly increases with $f_{0}$.

Therefore, a simple laser frequency modulation method for bi-axial scanning optical system can be proposed as follows:

(6) $$L_{M2}=N_{2}\frac{f_{x}}{f_{0}}=N_{2}n_{x}$$

where $N_{2}$ is a natural number and $L_{M2}$ is the laser modulation frequency. The slow-axis of the MEMS mirror was always operating to meet the Equation (6) that generated fringe patterns with different densities for dynamic pattern control. As shown in (2) of Mode 2 in Figure 9c, unlike the fringe patterns generated by the single-axial scanning optical system, the fringe patterns here were generated by Lissajous trajectory essentially, which enables the optical system to realize more complex patterns with the function of lighting and interactive projection in any interested area based on the refined time-sequence modulation of laser pulse and control circuit within allowable the field of view (FOV). In this test, the calculation of the number of fringes ($N_{f2}$) follows the similar principle of Equation (4):

(7) $$N_{f2}=\{\begin{array}{ll} N_{2}, & N_{2}=2n+1\\ N_{2}/2+1, & N_{2}=4n\\ N_{2}/2, & N_{2}=others \end{array}$$

when the $N_{2}$ = 8 in Equation (7), the dynamic light patterns can be generated by bi-axial scanning prototype optical system and algorithm simulation are shown in mode 2 of Figure 9c. The captured patterns all show that $N_{f2}$ = 5 is the same as the theoretically calculated value in Equation (7).

The performance comparison of the 1D and 2D Lissajous scanning mode for laser headlight prototype optical system is depicted in Figure 9. The electrical control principle of the 1D MEMS mirror is simple with an achievable FOV of 12.90°, but it requires an extra beam expander to generate 2D patterns. It is therefore only suitable for large FOV but simple scenes in a real traffic situation to provide basic lighting and illumination in limited degree-of-freedom of pattern control. In contrast, the optical system using 2D Lissajous MEMS scanning possesses higher illumination uniformity, more efficient optical paths, and simpler system architecture. Particularly, it enables arbitrary illumination areas with tunable and complex projection patterns in allowable FOV (12.54°), providing a preferred solution for realizing smart headlights with sophisticated reconfigurable light distribution functions. The $\theta_{opt}$ of the MEMS mirror meets the FOV requirements of headlights by considering two MEMS headlight modules that enable a total 25° light projection FOV and ensure three road lanes coverage over 30 m far-field distance, as shown in Figure 1. In addition, the proposed laser headlight prototype was compared with other existing headlight prototypes, as shown in Table 1. The MEMS LBS proposed in this paper achieves a larger mirror aperture size than the counterpart in reference [5], which is able to deliver 1.8-times higher optical power while considering the power carried by a MEMS scanner is proportional to the square of the mirror diameter [21]. Moreover, larger mirror size results in more refined resolvable projection pixels and thus higher resolution. Both fast- and slow-axis have been designed to operate at high-frequency, which allows for higher first eigenmode frequency than that in the reported work and benefits by improving the reliability of the system by offsetting the sensitive frequency away from vehicle vibration spectrum. Compared with the LCoS and DMD, the MEMS scanner provides much better optical efficiency. The much simpler requirements of module thermal design and fewer back-end lens arrays help to greatly reduce the costs.

## 4. Conclusions

In this paper, an automotive adaptive laser headlight prototype based on MEMS LBS with high optical efficiency was presented. A biaxial resonant MEMS mirror was piezoelectrically actuated to create 2D Lissajous laser patterns and project forward through phosphorous conversion of blue laser for structured illumination. The proposed MEMS design supports a large mirror size of 2 mm in diameter, which facilitates more refined resolvable pixels and delivers 1.8-times higher optical power than that in reported work. This design also meets the large aperture requirement of LiDAR and enables a promising all-in-one integration of ADB and LiDAR within single compact modules by sharing a common MEMS mirror as the spatial light modulator. The carefully designed bi-axial high-frequency resonance of Lissajous MEMS scanning improves the device reliability by offsetting the lowest eigenmode of mirror away from the vehicle random vibration. For demonstration, a simplified laser headlight prototype system was built, and the modulated illumination patterns based on 1D and 2D Lissajous scanning modes were evaluated, respectively. The tunability of scanning line density and shape size of Lissajous pattern were demonstrated by actual MEMS laser patterns projection and algorithm simulations, while a mathematical model of laser modulation and MEMS electrical control principle of Lissajous scan was established. It laid the foundation for developing a MEMS LBS control system for more complex pattern projections and prompting the application of MEMS SLMs for a compact ADB system that addresses nighttime driving visibility, eliminates glare effect, and renders interactive projection capabilities.

## Figures and Tables

**Table 1:** Performance comparison of different automotive headlight prototypes.

	MEMS Scanner (This Work)	MEMS Scanner Reference [5]	LCoS Reference [7]	DMD Reference [14]
Mirror aperture	2 mm	1.5 mm	N/A	N/A
Fast-axis frequency	17.328 kHz	21.29 kHz	N/A	N/A
Slow-axis frequency	4.811 kHz	60 Hz	N/A	N/A
1st eigenmode	>4 kHz	1 kHz	N/A	N/A
Illuminance power	high	medium	low	medium
Optical efficiency	high	high	low	low
Back-end lens	one	one	three or four	three or four
Resolution	(Variable) VGA	(Variable) SVGA	XGA	WVGA
